# Supplementary material for: Assessment of Soybean Lodging Using UAV Imagery and Machine Learning
Source: Plants (Basel). 2023 Aug 8;12(16):2893. doi: 10.3390/plants12162893 (PMC10458648; doi:10.3390/plants12162893)
Supplement: Supplementary file 1 [file plants-12-02893-s001.zip › plants-2505784-supplementary.pdf]

Supplementary Files

Table S1. Confusion matrix and model performance metrices of four lodging classes of soybean using original dataset and four machine learning (XGBoost, RF, KNN, and ANN) classifiers.

Classifier	Actual samples	NL	ML	HL	SL	Precision	Recall	F1-score
XGBoost	NL	201	1	0	0	0.80	1.00	0.89
	ML	35	1	0	0	0.25	0.03	0.05
	HL	10	2	0	0	0.00	0.00	0.00
	SL	4	0	0	0	0.00	0.00	0.00
RF	NL	200	2	0	0	0.80	0.99	0.88
	ML	35	1	0	0	0.25	0.03	0.05
	HL	11	1	0	0	0.00	0.00	0.00
	SL	4	0	0	0	0.00	0.00	0.00
KNN	NL	193	8	1	0	0.80	0.96	0.87
	ML	34	2	0	0	0.18	0.06	0.09
	HL	11	1	0	0	0.00	0.00	0.00
	SL	4	0	0	0	0.00	0.00	0.00
ANN	NL	186	4	3	0	0.75	0.96	0.84
	ML	35	0	0	0	0.00	0.00	0.00
	HL	21	1	0	0	0.00	0.00	0.00
	SL	4	0	0	0	0.00	0.00	0.00

Table S2. Confusion matrix and model performance metrices of four lodging classes of soybean using XGBoost classifier and SMOTE-Tomek Links, Borderline-SMOTE, SMOTE-NC, and ADASYN.

Pre-processed Dataset	Actual samples	NL	ML	HL	SL	Precision	Recall	F1-score
SMOTE-Tomek Link	NL	123	30	20	14	0.77	0.66	0.71
	ML	21	166	10	4	0.82	0.83	0.82
	HL	13	5	181	1	0.85	0.91	0.88
	SL	2	1	1	171	0.90	0.98	0.94
Borderline-SMOTE	NL	135	20	15	6	0.77	0.77	0.77
	ML	29	183	3	2	0.88	0.84	0.86
	HL	8	4	179	0	0.91	0.94	0.92
	SL	3	2	0	183	0.96	0.97	0.97
SMOTE-NC	NL	135	27	12	1	0.82	0.77	0.80

	ML	22	184	4	1	0.86	0.87	0.86
	HL	7	4	179	0	0.92	0.94	0.93
	SL	0	0	0	196	0.99	1.00	0.99
ADASYN	NL	122	30	23	6	0.80	0.67	0.73
	ML	20	160	11	4	0.80	0.82	0.81
	HL	11	9	179	1	0.84	0.90	0.86
	SL	0	0	1	179	0.95	0.99	0.97

Table S3. Confusion matrix and model performance metrices of four lodging classes of soybean using RF classifier and SMOTE-Tomek Links, Borderline-SMOTE, SMOTE-NC, and ADASYN.

Pre-processed Dataset	Actual samples	NL	ML	HL	SL	Precision	Recall	F1-score
SMOTE-Tomek Link	NL	121	34	19	13	0.81	0.65	0.72
	ML	16	174	6	5	0.82	0.87	0.84
	HL	9	4	184	3	0.87	0.92	0.90
	SL	4	0	2	169	0.89	0.97	0.93
Borderline-SMOTE	NL	134	22	16	4	0.78	0.76	0.77
	ML	25	187	5	0	0.88	0.86	0.87
	HL	10	4	177	0	0.89	0.93	0.91
	SL	3	0	0	185	0.98	0.98	0.98
SMOTE-NC	NL	125	26	18	6	0.80	0.71	0.76
	ML	22	179	7	3	0.85	0.85	0.85
	HL	7	6	172	5	0.87	0.91	0.89
	SL	2	0	1	193	0.93	0.98	0.96
ADASYN	NL	118	34	22	7	0.80	0.65	0.72
	ML	19	161	12	3	0.79	0.83	0.80
	HL	9	9	181	1	0.84	0.91	0.87
	SL	2	1	0	195	0.95	0.98	0.97

Table S4. Confusion matrix and model performance metrices of four lodging classes of soybean using KNN classifier and SMOTE-Tomek Links, Borderline-SMOTE, SMOTE-NC, and ADASYN.

Pre-processed Dataset	Actual samples	NL	ML	HL	SL	Precision	Recall	F1-score

SMOTE-Tomek Link	NL	64	60	36	27	0.89	0.34	0.49
	ML	6	173	15	7	0.72	0.86	0.78
	HL	1	7	189	3	0.78	0.94	0.86
	SL	1	1	2	171	0.82	0.98	0.89
Borderline-SMOTE	NL	91	48	28	9	0.79	0.52	0.63
	ML	18	191	5	3	0.77	0.88	0.82
	HL	5	9	177	0	0.84	0.93	0.88
	SL	1	1	1	185	0.94	0.98	0.96
SMOTE-NC	NL	84	55	24	12	0.74	0.48	0.58
	ML	18	172	17	4	0.72	0.82	0.76
	HL	10	12	164	4	0.77	0.86	0.81
	SL	2	1	9	184	0.90	0.94	0.92
ADASYN	NL	61	51	49	20	0.80	0.34	0.47
	ML	9	170	15	1	0.72	0.87	0.79
	HL	6	12	181	1	0.73	0.91	0.81
	SL	0	2	2	194	0.90	0.98	0.98

Table S5. Confusion matrix and model performance metrices of four lodging classes of soybean using ANN classifier and SMOTE-Tomek Links, Borderline-SMOTE, SMOTE-NC, and ADASYN.

Pre-processed Dataset	Actual samples	NL	ML	HL	SL	Precision	Recall	F1-score
SMOTE-Tomek Link	NL	105	58	24	5	0.55	0.87	0.67
	ML	14	162	8	4	0.86	0.72	0.78
	HL	2	6	193	1	0.95	0.86	0.90
	SL	0	0	0	181	1.00	0.95	0.97
Borderline-SMOTE	NL	133	56	21	0	0.63	0.90	0.74
	ML	7	171	2	0	0.95	0.74	0.83
	HL	6	3	191	0	0.95	0.89	0.92
	SL	1	1	0	180	0.98	1.00	0.99
SMOTE-NC	NL	166	27	15	2	0.79	0.82	0.81
	ML	21	149	10	0	0.83	0.81	0.82
	HL	14	8	177	1	0.88	0.87	0.88
	SL	0	0	0	182	1.00	0.98	0.99

ADASYN	NL	94	59	40	5	0.48	0.85	0.61
	ML	12	166	9	1	0.88	0.72	0.79
	HL	4	4	184	5	0.93	0.78	0.85
	SL	0	0	0	191	1.00	0.94	0.97

Table S6: Description of the hyperparameters used for the analysis using XGBoost Classifier.

Hyper-parameters	Description
n_estimators	The number of boosting rounds or decision tree to be built.
max_depth	The maximum depth of each decision tree.
learning_rate	The step size shrinkage used in updating weights during each boosting round. A lower learning rate can help prevent overfitting but may require a higher number of boosting rounds.
gamma	The minimum loss reduction required to split a node. A higher value can lead to fewer and more conservative splits, while a lower value can lead to more splits and potentially overfitting.
subsample	The fraction of observations to be randomly sampled for each tree. A lower value can lead to a more conservative model, while a higher value can lead to overfitting.
colsample_bytree	The fraction of columns to be randomly sampled for each tree. A lower value can lead to a more conservative model, while a higher value can lead to overfitting.
min_child_weight	The minimum sum of instance weight needed in a child. A higher value can lead to a more conservative model, while a lower value can lead to overfitting.

Table S7: XGBoost hyper-parameter best values using TPE method for the analysis of original dataset and treated dataset (SMOTE-Tomek Links, SMOTE-ENN, Borderline-SMOTE, SMOTE-NC and ADASYN) for balancing.

Hyper-parameter	Search Space	Best Values					
		Original dataset	Dataset Treated by SMOTE-Tomek Link	Dataset Treated by SMOTE-ENN	Dataset Treated by Borderline-SMOTE	Dataset Treated by SMOTE-NC	Dataset Treated by ADASYN
n_estimators	100, 1000	348	593	337	664	944	652
max_depth	3, 10	6	8	10	10	8	9
learning_rate	0.01, 1	0.01	0.08	0.08	0.03	0.06	0.09
gamma	0, 1	0.56	0.20	0.03	0.003	0.19	0.13
subsample	0.5, 1	0.99	0.62	0.8	0.80	0.8	0.71

colsample_bytree	0.5, 1	0.79	0.90	0.98	0.83	0.9	0.76
min_child_weight	1, 5	2	1	2	2	1	1

Table S8: Description of the hyperparameters used for the analysis using RF Classifier.

Hyper-parameters	Description
n_estimators	Determines the number of decision trees to be built in the random forest.
max_features	Identifies the maximum number of features needed when splitting a node in each decision tree. It controls the randomness in feature selection.
min_samples_split	This hyperparameter determines the minimum number of samples needed to split an internal node in a decision tree.
min_samples_leaf	Identifies the minimum number of samples required to be at a leaf node in a decision tree.
Max_depth	Identifies the number of maximum depths of a decision tree which mainly limits the number of levels in the tree, prevents overcomplexity, and reduce the overfitting.
bootstrap	Determines the bootstrap samples were used or not to build each decision tree in the random forest.

Table S9: RF hyper-parameter best values using grid search method for the analysis of original dataset and treated dataset (SMOTE-Tomek Links, SMOTE-ENN, Borderline-SMOTE, SMOTE-NC and ADASYN) for balancing.

Hyper-parameter	Search Space	Best Values					
		Original dataset	Dataset Treated by SMOTE-Tomek Link	Dataset Treated by SMOTE-ENN	Dataset Treated by Borderline-SMOTE	Dataset Treated by SMOTE-NC	Dataset Treated by ADASYN
n_estimators	50, 100, 200	50	50	100	100	100	50
max_features	sqrt, log2	sqrt	sqrt	sqrt	sqrt	sqrt	sqrt
min_samples_split	1, 2, 4	1	2	1	1	1	1
min_samples_leaf	1, 10	5	5	5	10	5	5
Max_depth	1, 10, 50	10	10	10	10	10	10
bootstrap	True, False	True	True	True	True	True	True

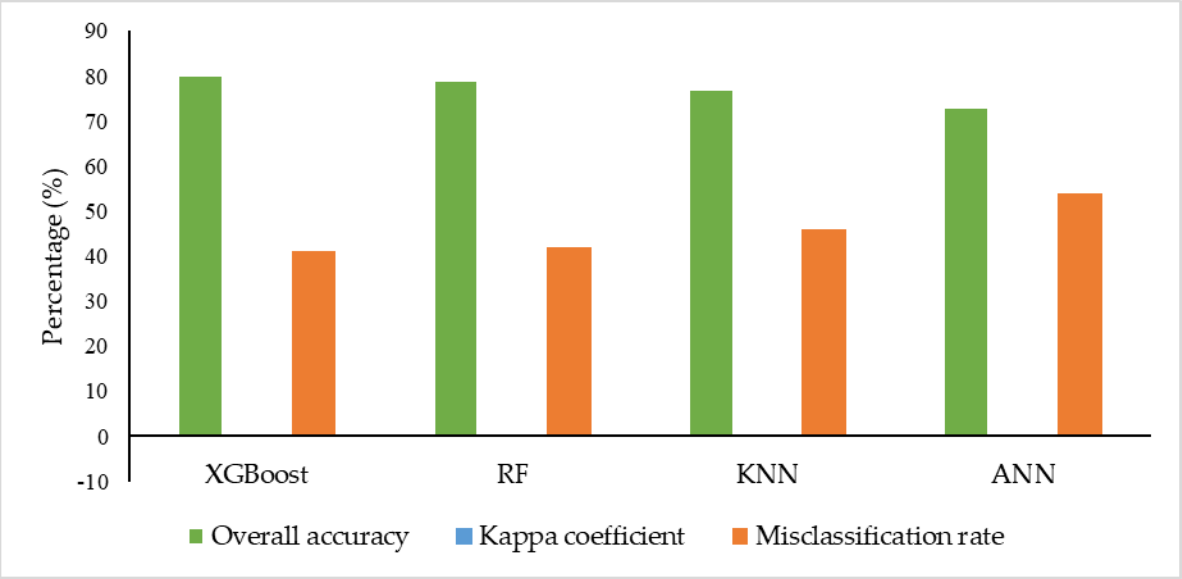

Figure S1. Overall accuracy, kappa coefficient and misclassification rate achieved using four original imbalanced dataset and four machine learning classifiers.

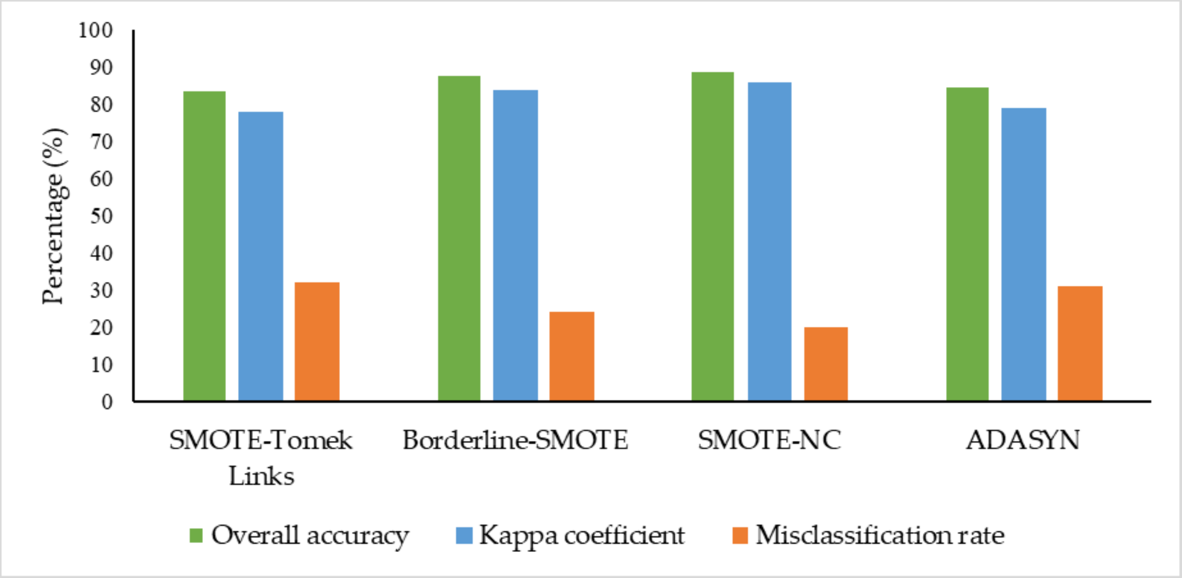

Figure S2. Overall accuracy, kappa coefficient and misclassification rate achieved using four (SMOTE-Tomek Links, Borderline-SMOTE, SMOTE-NC, and ADASYN) balanced dataset and XGBoost classifier.

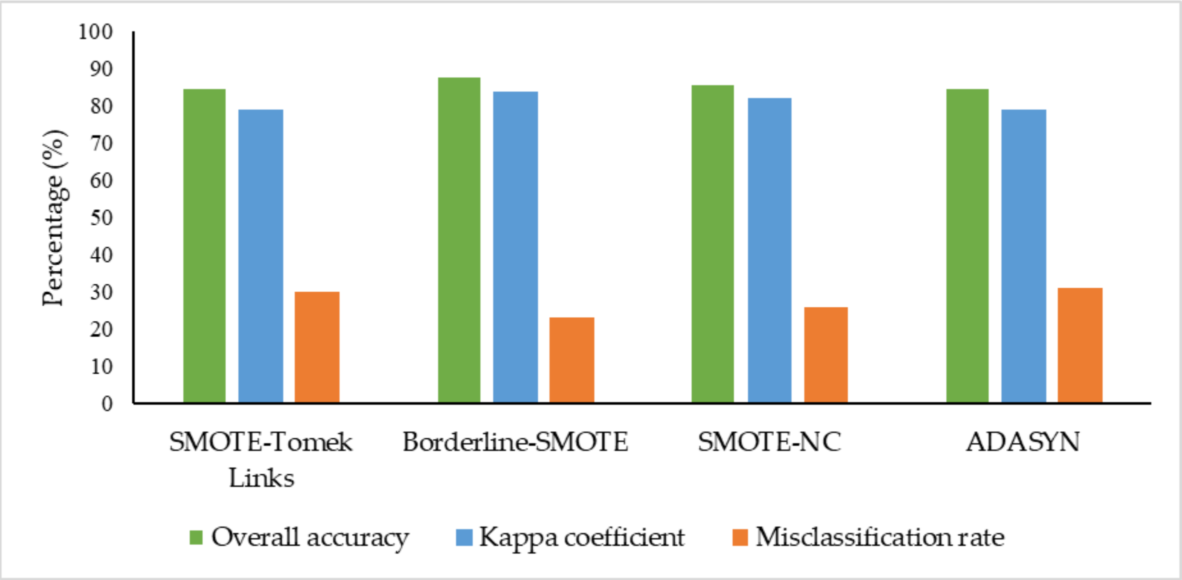

Figure S3. Overall accuracy, kappa coefficient and misclassification rate achieved using four (SMOTE-Tomek Links, Borderline-SMOTE, SMOTE-NC, and ADASYN) balanced dataset and RF classifier.

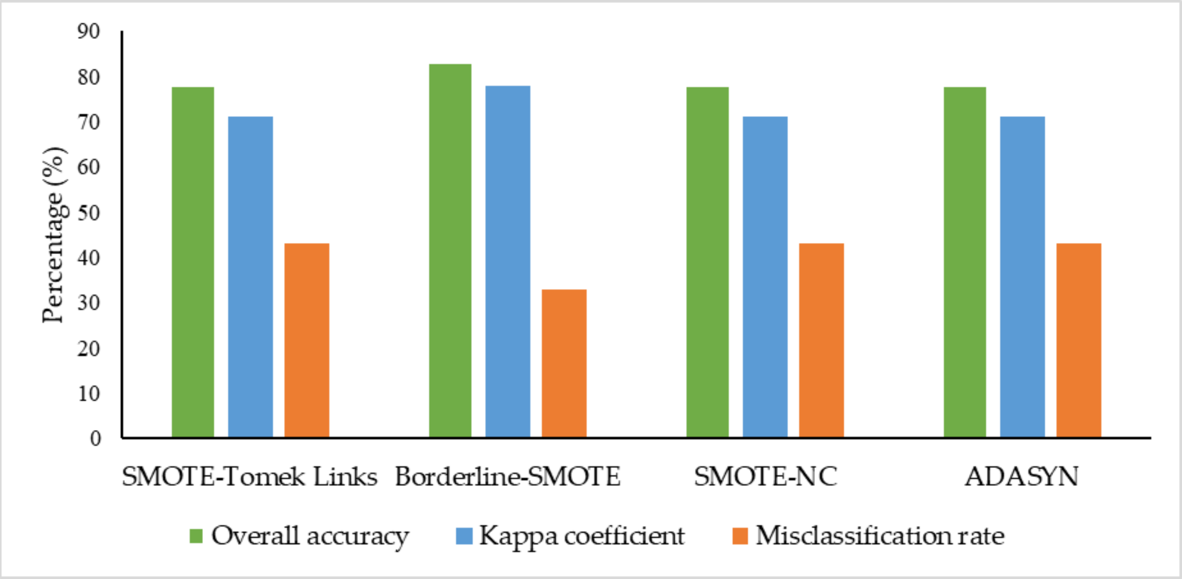

Figure S4. Overall accuracy, kappa coefficient and misclassification rate achieved using four (SMOTE-Tomek Links, Borderline-SMOTE, SMOTE-NC, and ADASYN) balanced dataset and KNN classifier.

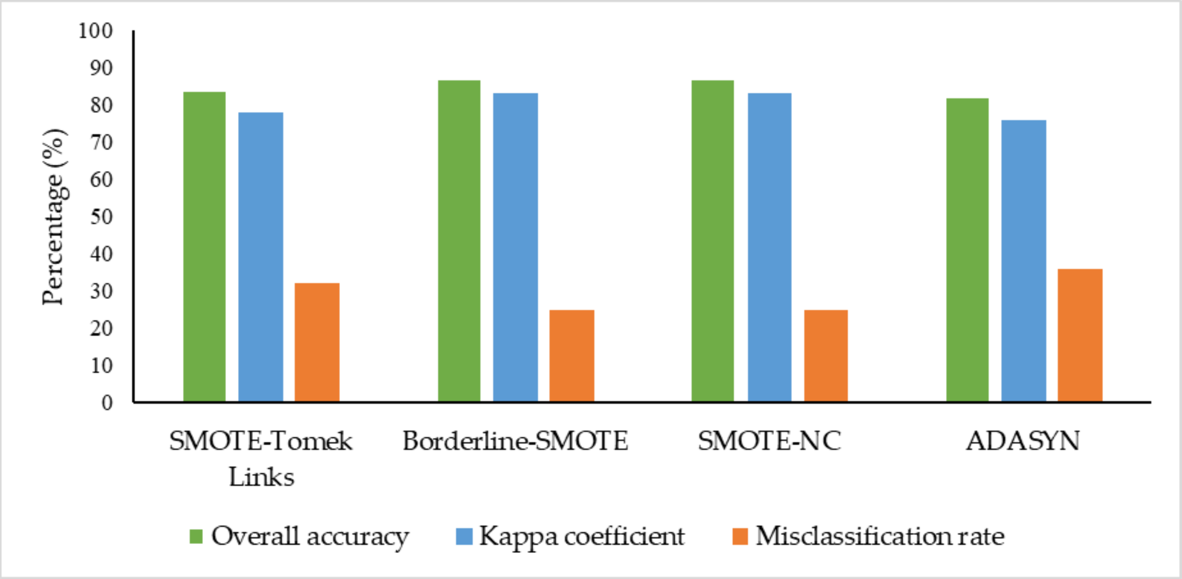

Figure S5. Overall accuracy, kappa coefficient and misclassification rate achieved using four (SMOTE-Tomek Links, Borderline-SMOTE, SMOTE-NC, and ADASYN) balanced dataset and ANN classifier.

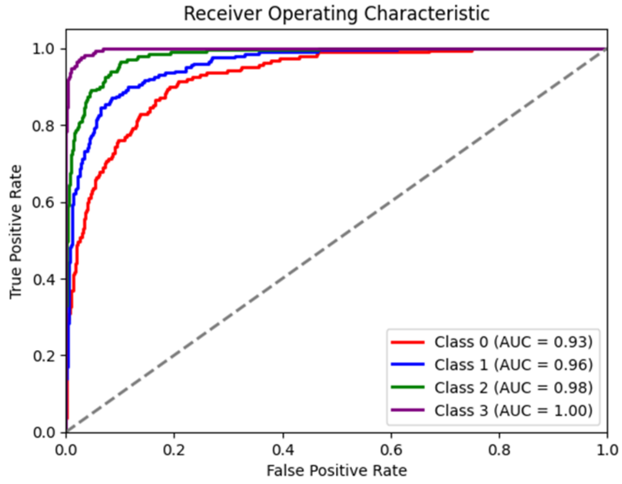

(a) SMOTE-Tomek Links

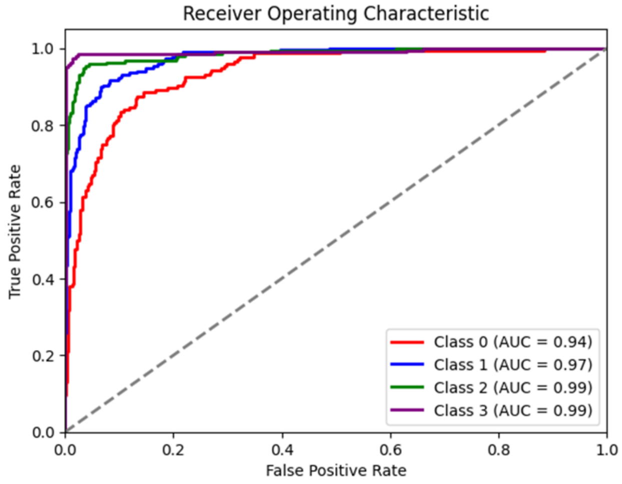

(b) Borderline-SMOTE

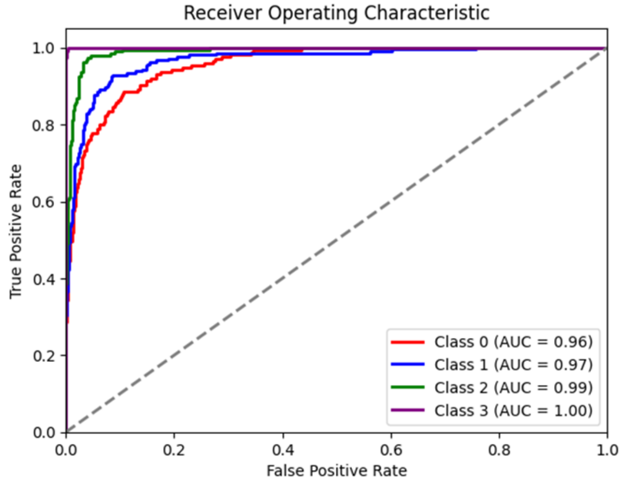

(c) SMOTE-NC

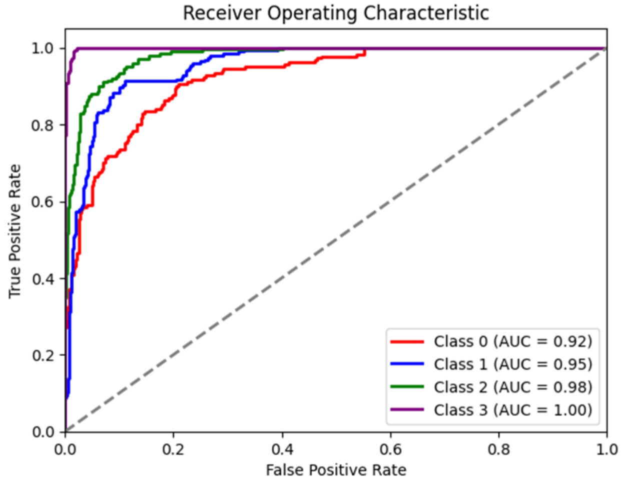

(d) ADASYN

Figure S6. The ROC curve using four (SMOTE-Tomek Links, Borderline-SMOTE, SMOTE-NC, and ADASYN) balanced datasets and XGBoost classifier**.**

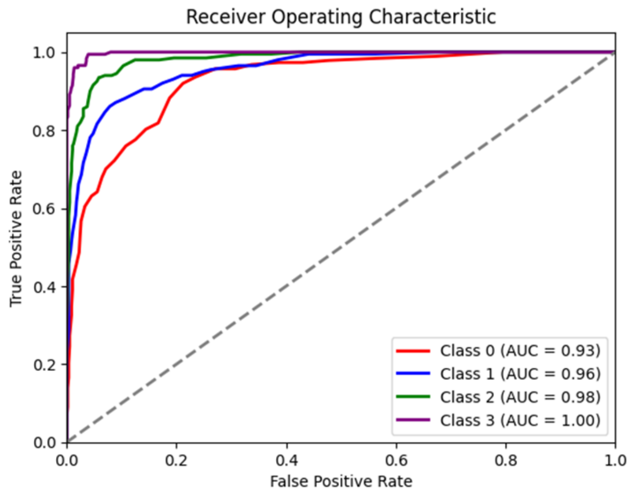

(a) SMOTE-Tomek Links

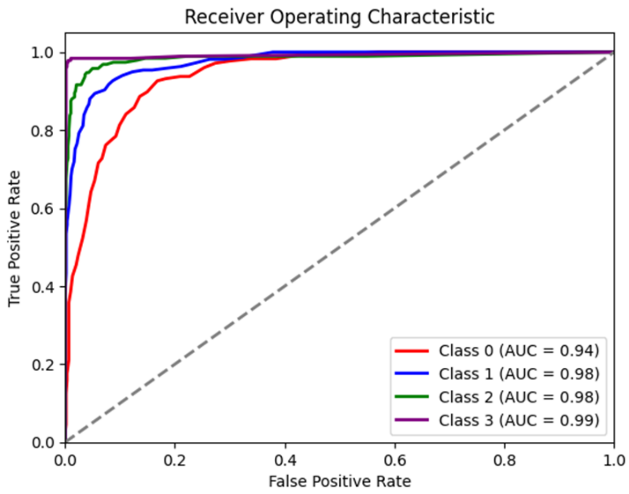

(b) Borderline-SMOTE

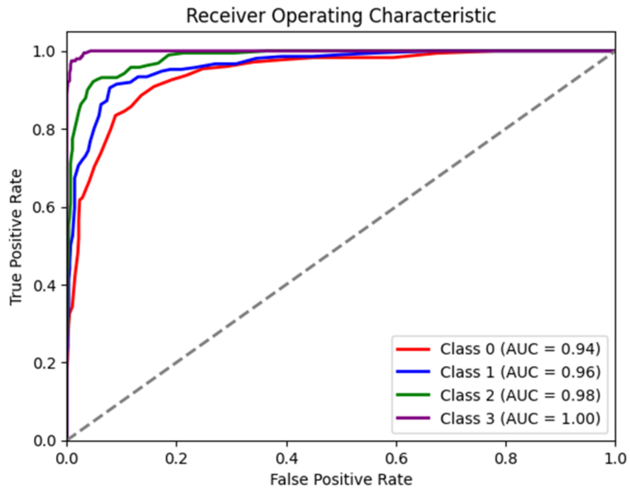

(c) SMOTE-NC

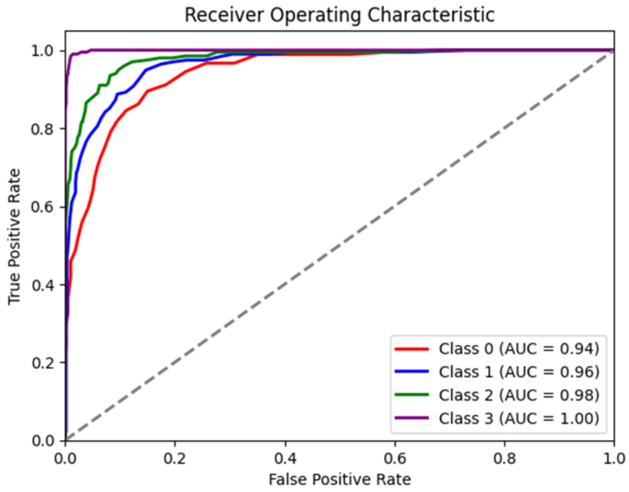

(d) ADASYN

Figure S7. The ROC curve using four (SMOTE-Tomek Links, Borderline-SMOTE, SMOTE-NC, and ADASYN) balanced datasets and RF classifier**.**

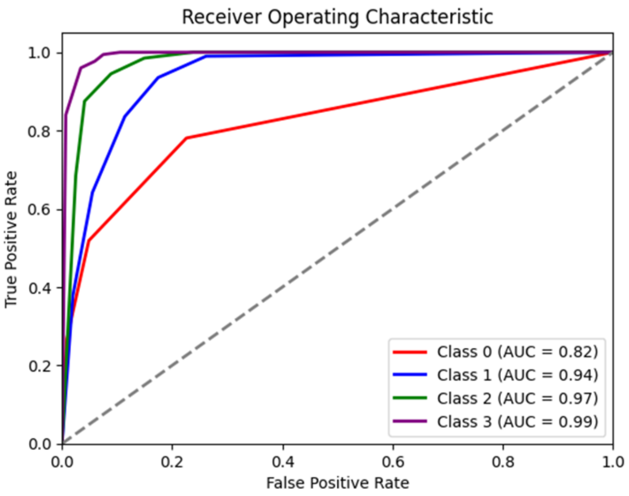

(a) SMOTE-Tomek Links

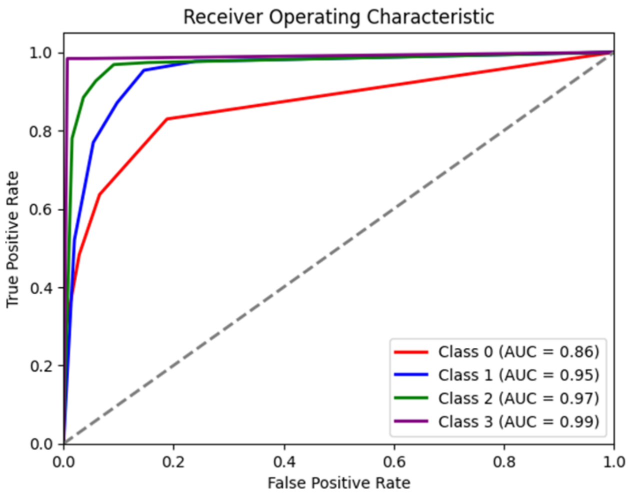

(b) Borderline-SMOTE

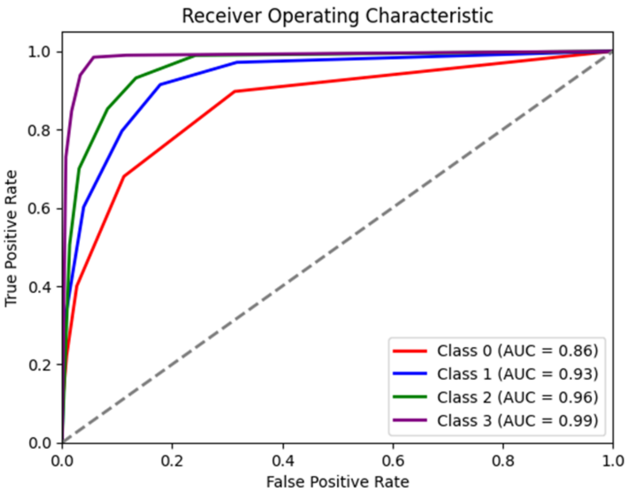

(c) SMOTE-NC

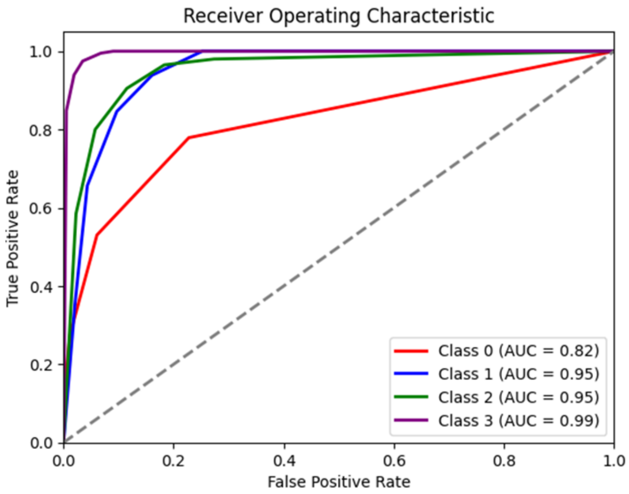

(d) ADASYN

Figure S8. The ROC curve using four (SMOTE-Tomek Links, Borderline-SMOTE, SMOTE-NC, and ADASYN) balanced datasets and KNN classifier.

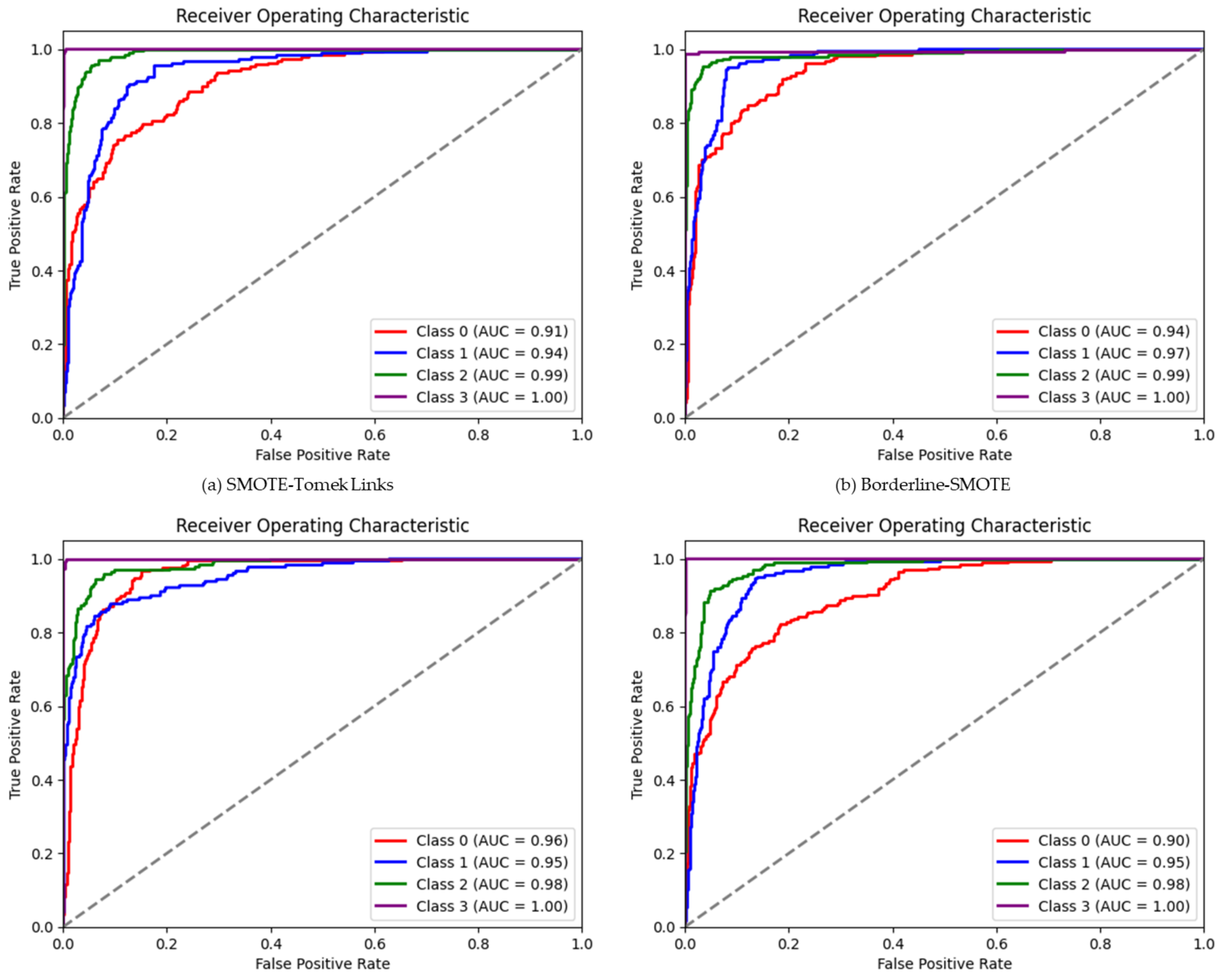

(a) SMOTE-Tomek Links

(b) Borderline-SMOTE

(c) SMOTE-NC

(d) ADASYN

Figure S9. The ROC curve using four (SMOTE-Tomek Links, Borderline-SMOTE, SMOTE-NC, and ADASYN) balanced datasets and ANN classifier**.**

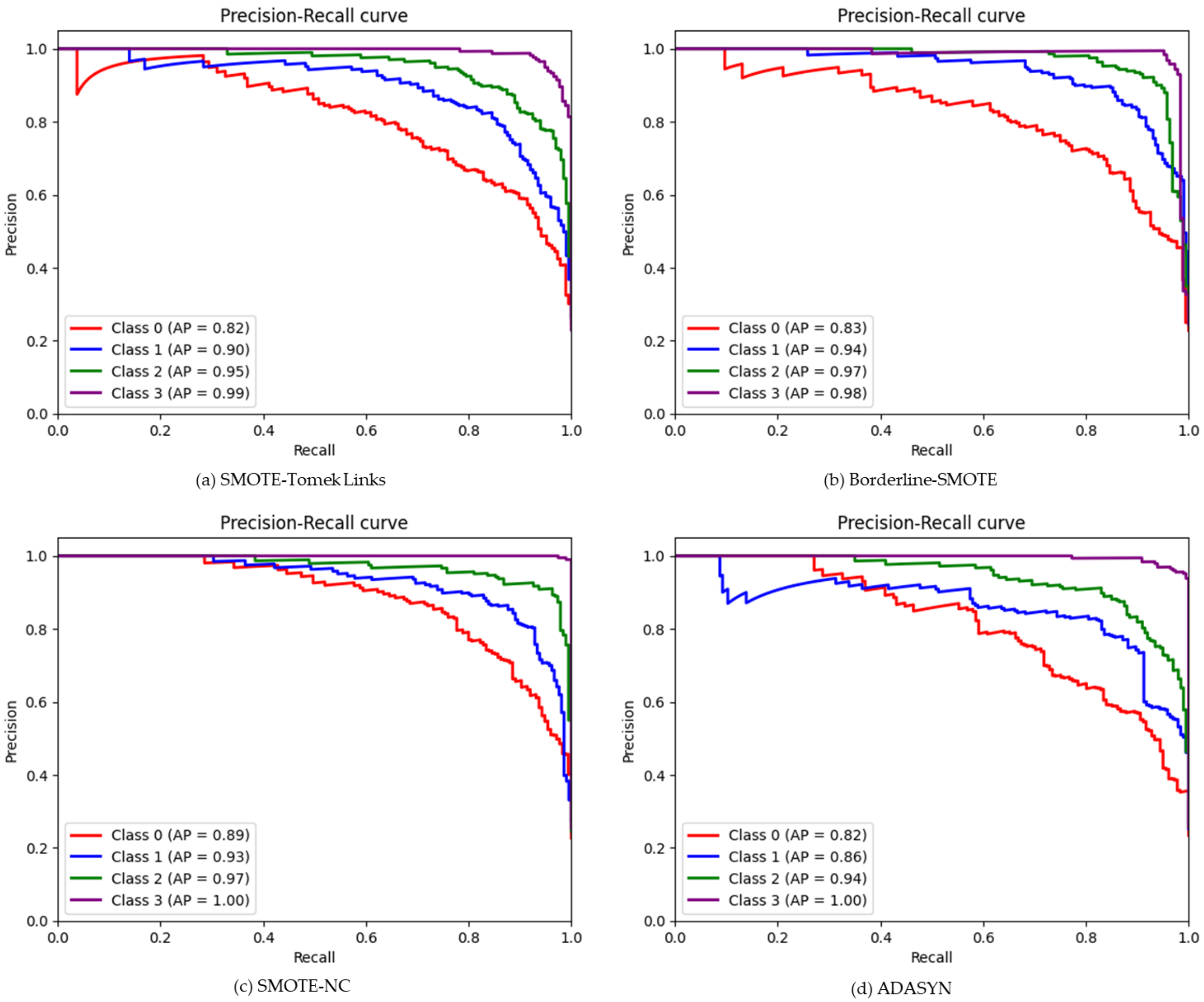

(a) SMOTE-Tomek Links

(b) Borderline-SMOTE

(c) SMOTE-NC

(d) ADASYN

Figure S10. The Precision-Recall curve using four (SMOTE-Tomek Links, Borderline-SMOTE, SMOTE-NC, and ADASYN) balanced datasets and XGBoost classifier.

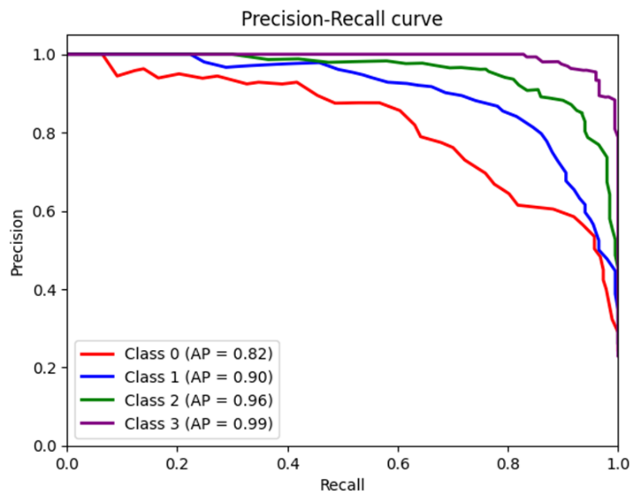

(a) SMOTE-Tomek Links

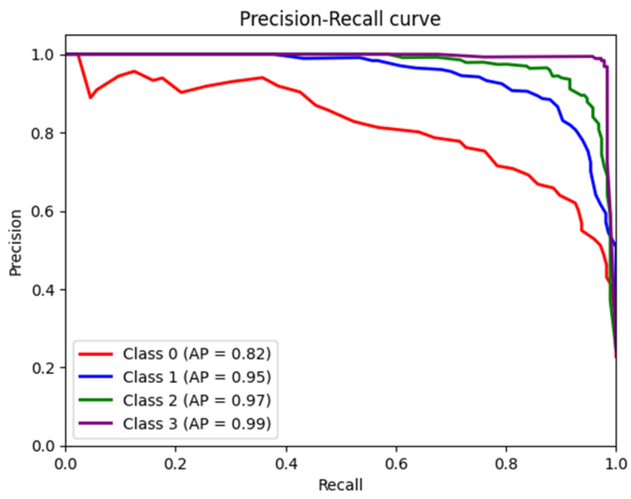

(b) Borderline-SMOTE

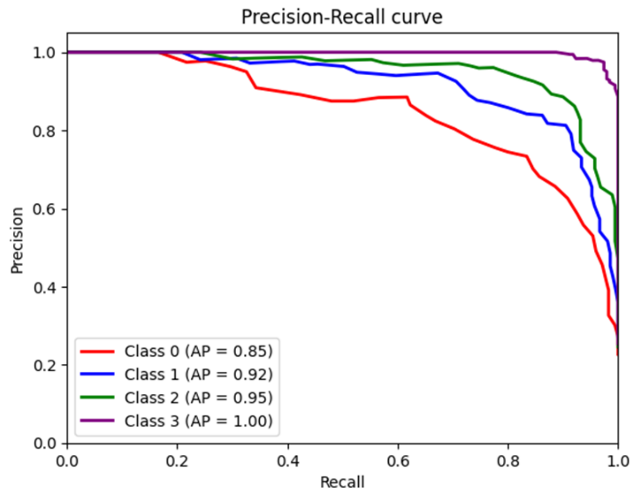

(c) SMOTE-NC

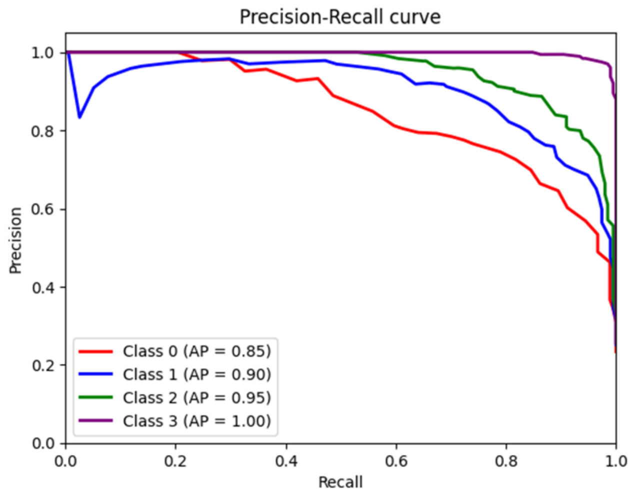

(d) ADASYN

Figure S11. The Precision-Recall curve using four (SMOTE-Tomek Links, Borderline-SMOTE, SMOTE-NC, and ADASYN) balanced datasets and RF classifier.

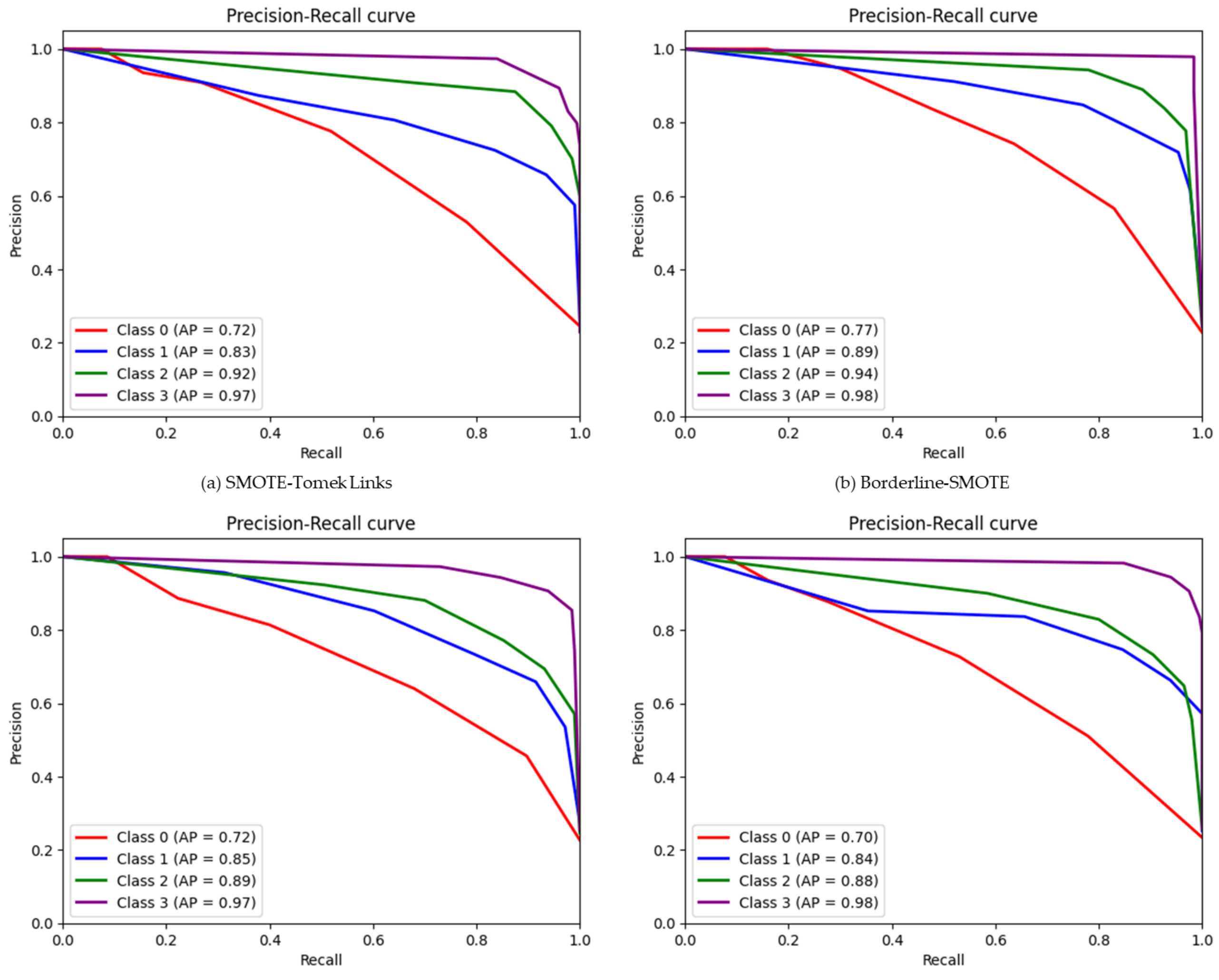

(a) SMOTE-Tomek Links

(b) Borderline-SMOTE

(c) SMOTE-NC

(d) ADASYN

Figure S12. The Precision-Recall curve using four (SMOTE-Tomek Links, Borderline-SMOTE, SMOTE-NC, and ADASYN) balanced datasets and KNN classifier.

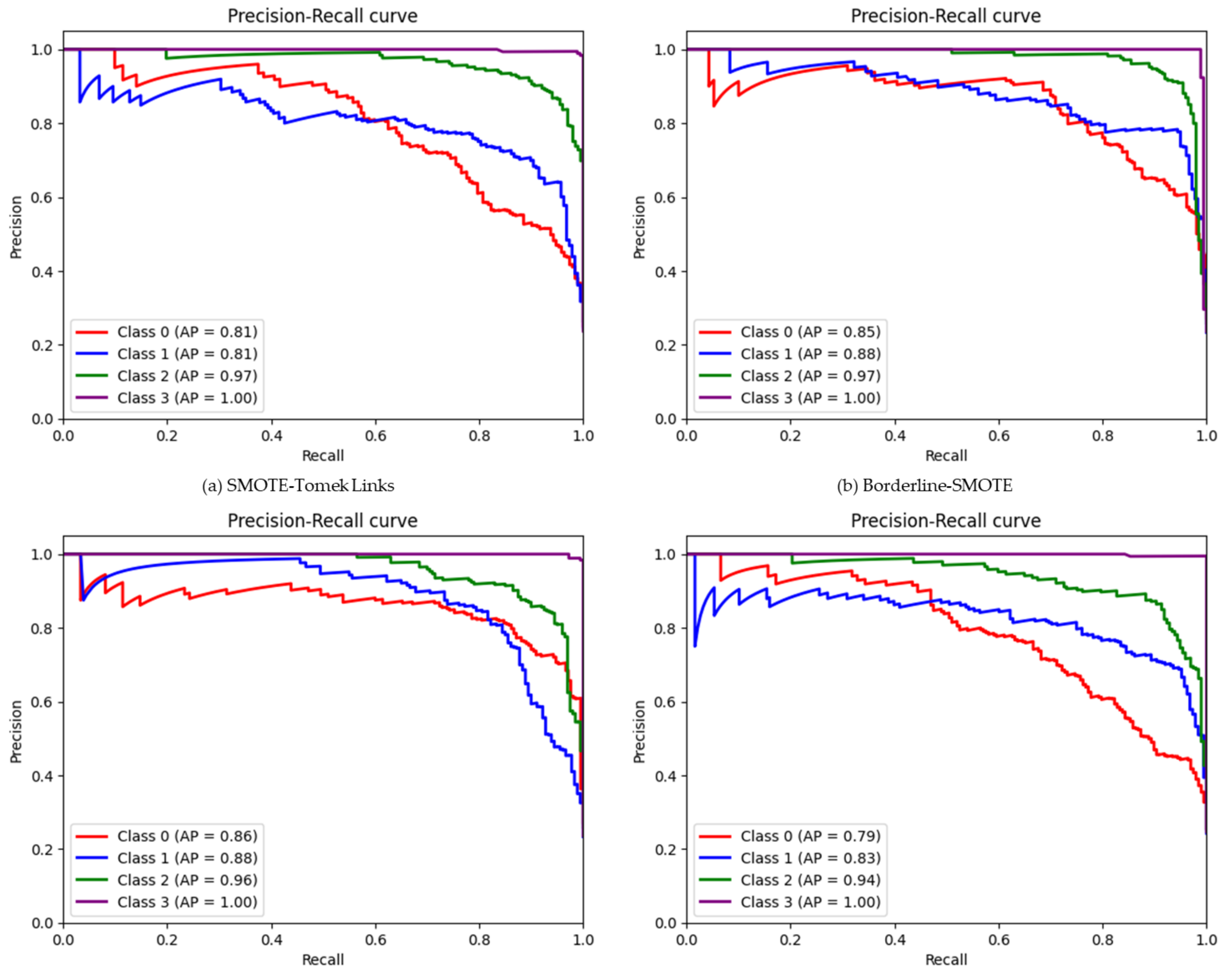

(a) SMOTE-Tomek Links

(b) Borderline-SMOTE

(c) SMOTE-NC

(d) ADASYN

Figure S13. The Precision-Recall curve using four (SMOTE-Tomek Links, Borderline-SMOTE, SMOTE-NC, and ADASYN) balanced datasets and ANN classifier.
